# Sleep and physical activity patterns in adults and children with Bardet–Biedl syndrome

**DOI:** 10.1186/s13023-021-01911-4

**Published:** 2021-06-14

**Authors:** Jeremy Pomeroy, Jeffrey J. VanWormer, Jill R. Meilahn, Tara Maki, Hema R. Murali, Robert M. Haws

**Affiliations:** 1 Department of Clinical Research, Marshfield Clinic Research Institute, 1000 North Oak Ave, Marshfield, WI 54449 USA; 2 Clinical Epidemiology and Population Health, Marshfield Clinic Research Institute, Marshfield, WI USA; 3 Department of Physical Medicine, Marshfield Clinic Health System, Marshfield, WI USA; 4 Physical Therapy Department, Marshfield Clinic Health System, Marshfield, WI USA; 5 Department of Pediatrics, Marshfield Clinic Health System, Marshfield, WI USA

**Keywords:** Bardet–Biedl syndrome, Sleep, Sedentary time, Physical activity, Accelerometer

## Abstract

**Background:**

Overweight and obesity are common features of the rare disease Bardet–Biedl syndrome (BBS). Sleep and physical activity are behaviors that might impact overweight and obesity and thus may play a key role in the health and well-being of people with BBS. Objectively-measured sleep and physical activity patterns in people with BBS are not well known. We evaluated objectively-measured sleep and physical activity patterns in the largest cohort to date of people with BBS.

**Results:**

Short sleep duration, assessed using wrist-worn accelerometers, was common in both children and adults with BBS. Only 7 (10%) of adults and 6 (8%) of children met age-specific sleep duration recommendations. Most adults 64 (90%) achieved recommended sleep efficiency. The majority of children 26 (67%) age 6–12 years achieved recommended sleep efficiency, but among children age 13–18, only 18 (47%). In both adults and children, sleep duration was significantly negatively correlated with duration of prolonged sedentary time. In children age 6–12 sleep duration was also significantly related to total activity score, children with lower sleep duration had lower total activity scores.

**Conclusions:**

Insufficient sleep duration is very common in people with BBS. Prolonged sedentary time and short sleep duration are both potentially important health-related behaviors to target for intervention in people with BBS.

**Supplementary Information:**

The online version contains supplementary material available at 10.1186/s13023-021-01911-4.

## Background

Bardet–Biedl syndrome (BBS) is a rare ciliopathy with an estimated prevalence of 1 in 100,000 births, impacting approximately 3,500 individuals in the United States [1]. Primary clinical features of BBS include obesity, renal anomalies, retinal degeneration, and learning disabilities, which often lead to diabetes, blindness, or chronic kidney disease in adulthood.

Obesity is perhaps the most common visible feature of BBS, and is clearly among the most concerning to parents of children with BBS [2]. Excess body weight gain is rapid and is observed very early in patients with BBS, with rates of overweight/obesity exceeding 90% after age 5 and persisting through adolescence (and presumably adulthood) [3]. A small study of people with BBS found that sleep disordered breathing is common [4], with 18 of the 20 participants having sleep difficulties. Objectively-measured physical activity behavior has previously been characterized in a small cohort of people with BBS [5]. People with BBS were found to have a lower total activity level than age-, sex-, and body mass index (BMI)-matched controls. Little is known about the benefits of sleep and physical activity in people with BBS, but in other populations adequate sleep and higher levels of physical activity and lower levels of sedentary time are associated with protection against weight gain [6, 7] as well as a protective impact on negative health impacts of obesity [8, 9].

There are no known prior studies that have objectively measured both sedentary and sleep time in people with BBS. These are potentially critical behaviors in the prevention of obesity and its negative health impacts, which is a chief medical and social concern for patients with BBS. A richer understanding of sleep and physical activity in people with BBS will help identify opportunities for more targeted, comprehensive behavioral approaches to mitigate excess weight gain, or promote weight loss, in this rare disease population where obesity is ubiquitous. The purpose of this study was to characterize objectively measured physical activity and sleep patterns and associations between objectively measured physical activity and sleep patterns in the largest cohort of people with BBS.

## Methods

### Participants

The target population was participants in the Clinical Registry Investigating Bardet–Biedl Syndrome (CRIBBS), an international database designed to record health outcomes of individuals with BBS (ClinicalTrials.gov: NCT02329210). Participants in CRIBBS or their caregivers are asked to take part in annual health interviews carried out by a research coordinator. A substudy to evaluate sleep and activity behavior using a wrist-worn, high-resolution triaxial accelerometer was approved by the Marshfield Clinic Research Institute Institutional Review Board in January 2017. Participants enrolled in February 2017, and analyses were completed by June 2019. Eligible participants resided in the United States and were 3 years-of-age and older. Those individuals/legal guardians that have agreed to be contacted about BBS research opportunities were contacted by a research coordinator and informed of the study. Interested individuals were recruited for participation in the research via telephone during the annual CRIBBS interview, and an accelerometer was mailed to participants based on participant and monitor availability. Consent was provided verbally by the participant or their legal guardian.

### Measurement procedures

Physical activity and sleep behaviors were assessed using the ActiGraph GT9X Link wrist-worn, high-resolution triaxial accelerometer (ActiGraph Corp., Pensacola, FL). The Link is a 3.5 × 3.5 × 1 cm device weighing 14 g. The Link contains a solid state 3-axis MEMS accelerometer with a ± 8 g dynamic range and is capable of recording time-stamped gravitational acceleration at a sample rate of 30–100 Hz.

The Link was initialized to record at a 30 Hz sample rate and set to record for 12 days. Participants were asked to wear the monitor at all times with the exception of bathing. The Link monitors were mailed to participants with a padded, pre-addressed, postage-paid envelope to return the device. The monitors were initialized with a delayed start time to account for time the Link was in transit to the participant. Once the Link was returned, data were downloaded to a secure hard-drive. Participants with at least 4 days of wear time were provided with a sleep report describing their average sleep duration and their frequency meeting their age-recommended sleep duration.

Data processing was performed using ActiLife software v6.13.4. Sleep data were processed using the Batch Sleep Feature, sedentary time was processed using the Sedentary Analysis Feature [10], and total physical activity was processed by outputting 1 s epoch data using ActiLife’s proprietary normal filter to CSV files and completing the processing in a program written in SAS. Sleep was scored using the Sadeh sleep algorithm [11] for participants age 19 and older and the Cole-Kripke sleep algorithm [12] for participants under age 19. We used the ActiLife-modified Tudor-Locke algorithm to identify sleep periods for all age groups. The time in bed is all time, both awake and asleep after the first 5 consecutive minutes of sleep and before 10 min of consecutive awake. Total sleep time is the time scored as sleep during in bed periods. Participants with less than 75% wear time for the monitoring period were omitted from the analysis.

### Outcomes

Sleep variables included sleep duration and continuity. Sleep duration was reported as hours in bed per day and total sleep hours per day. Sleep duration was also classified as meeting or exceeding recommendations for age [13, 14]. Sleep continuity included sleep efficiency and sleep fragmentation index. Sleep efficiency is the percentage of time in bed scored as sleep. Sleep fragmentation index, a measure of restlessness, corresponds to changes from deep sleep to light sleep, as assessed by sleep polysomnography [15]. Sleep fragmentation is expressed as a percentage of changes over total sleep time.

Awake sedentary time was determined using vector magnitude. The minimum sedentary bout time was set at 20 min with a 2 min drop time. Prolonged sedentary time included the median duration of all sedentary bouts lasting at least 20 min and the median daily duration of prolonged all sedentary time accumulated in bouts lasting at least 20 min as assessed during non-sleep periods over the 12-day measurement period.

Total physical activity was determined using normal-filtered triaxial vector magnitude data. The average daily vector magnitude per minute during non-sleep periods was expressed as a z-score referenced to the cohort, with a mean of 0 and a standard deviation of 1.

### Statistical analysis

Data were analyzed using SAS version 9.4 (SAS Institute Inc., Cary, NC, USA). Descriptive statistics were medians and 25th and 75th centiles. Participants were stratified by children/adolescents (age 6 to 18 years) and adults (age 19 and above). Spearman correlations were calculated between age and the sleep and physical activity variables, stratified by age categories. Spearman correlations between the sleep variables and physical activity variables were also stratified by age group. Age-stratified Wilcoxon tests were performed to compare physical activity between individuals meeting versus not meeting sleep recommendations for age. Two-tailed p-values were considered significant at < 0.05, without adjustment for multiple comparisons.

## Results

Descriptive statistics for participants are shown in Table 1. There were 391 participants in the CRIBBS registry who were eligible to participate. Of the 206 individuals invited, 29 declined (16 adults, 13 children), 16 participants (8 adults, 8 children) who agreed to wear monitors did not have sufficient wear time to be included in the analysis, and 8 monitors were lost. Only five children age 3–5 participated in the accelerometry study, and due to the small sample size, were excluded from the analysis. There were no significant differences for sleep or physical activity variables by sex in either adults or children (data not shown). Over one third of adults and a quarter of the children who had undergone a sleep study reported abnormal sleep study results. Nearly 30% of adults and 10% of children reported receiving treatment for sleep apnea.

**Table 1:** Descriptives

	Adults n = 71 (59% female)	All children n = 77 (47% female)	Children 6–12 years n = 39	Children 13–18 years n = 38
Age (years)*	32.9 (24.6, 39.6)	12.9 (10.9, 16.5)	10.9 (9.6, 11.8)	16.6 (14.8, 17.7)
Days of observation (days)*	11.6 (10.1, 11.8)	11.6 (10.5, 11.8)	11.6 (10.2, 11.8)	11.8 (10.7, 11.8)
Wear time (%)*	96 (83.6, 99.1)	91.8 (83.3, 97.9)	91.7 (78.4, 96.4)	92.85 (86.4, 98.5)
Median duration of prolonged sedentary bouts (min)*	32 (29, 37)	35.5 (29, 42)	37.5 (33, 45.5)	32.75 (28, 37)
Total prolonged sedentary bout time (min/day)*	225 (174.5, 282)	112 (86, 168)	106 (85, 159.5)	123.75 (87, 189)
Total activity Z-score (standard deviations)*	− 0.13 (− 0.86, 0.68)	− 0.09 (− 0.81, 0.45)	0.06 (− 1.22, 0.96)	− 0.18 (− 0.54, 0.29)
Time in Bed (h/day)*	5.4 (4.5, 6.5)	7.6 (6.3, 8.7)	8.4 (7.6, 9.0)	6.4 (5.5, 7.3)
Total sleep time (h/day)*	4.8 (3.9, 6.0)	6.7 (5.6, 7.9)	7.6 (6.7, 8.2)	5.9 (4.9, 6.7)
Sleep efficiency (%)*	89.89 (87.73, 92.74)	90.97 (87.4, 92.28)	91.28 (88.51, 92.6)	89.88 (86.9, 92.21)
Sleep fragmentation index (%)*	19.77 (14.39, 24.72)	20.27 (17.3, 23.18)	19.39 (16.06, 22.98)	20.73 (17.95, 23.49)
Abnormal sleep study (yes)**	24 (34.8)	18 (26.1)	10 (26.3)	8 (21.1)
Sleep apnea treatment (yes)**	19 (27.5)	7 (10.1)	3 (7.9)	4 (10.5)
Diabetes mellitus (yes)**	16 (10.5)	5 (3.3)	1 (1.2)	4 (4.9)
Suspected restless leg syndrome (yes)**	38 (25.9)	30 (20.4)	18 (23.7)	12 (15.8)

Statistics are median (25th, 75th) centiles* or number (percent) reporting yes**

The frequency of meeting or exceeding age-recommended sleep duration and sleep efficiency is shown in Table 2. Only 10% of adults and 8% of children met the recommended sleep duration for age. Conversely, 3% of adults and no children exceeded the upper limit of sleep duration for age. Only 8% in each the 6–12 years and 13–18 years age groups met the age-recommended sleep duration. While meeting the age-recommended sleep duration was rare, 90% of adults met the age-recommended sleep efficiency. Meeting the recommendation for sleep efficiency was less common among children, with 54% overall meeting the recommendation.

**Table 2:** Age group stratified frequency of meeting sleep recommendations

	Adults N (%)	All children N (%)	Children 6–12 years N (%)	Children 13–18 years N (%)
Meeting sleep duration recommendation for age	7 (10.1)	6 (7.8)	3 (7.7)	3 (7.9)
Exceeding sleep duration recommendation for age	2 (2.8)	0 (0)	0 (0)	0 (0)
Meeting sleep efficiency recommendation for age	64 (90.1)	44 (57.1)	26 (66.7)	18 (47.3)

Correlations between age and sleep or physical activity are shown in Table 3. No measures of sleep or physical activity were significantly correlated with age in adults. In children, sleep duration and time in bed were significantly positively correlated with age. Measures of sleep continuity were not significantly correlated with age in children. The average duration of prolonged sedentary bouts was significantly negatively correlated with age in children. The average daily duration of prolonged sedentary time was not significantly correlated with age in children.

**Table 3:** Age group stratified correlations with age

	Adults age (years)	Children age (years)
Median duration of prolonged sedentary bouts (min)	r = − 0.021 p = 0.86	**r = **− **0.39** **p = 0.0006**
Total prolonged sedentary bout time (min/day)	r = 0.20 p = 0.11	r = 0.13 p = 0.26
Total activity Z-score (standard deviations)	r = − 0.12 p = 0.32	r = − 0.21 p = 0.07
Time in bed (h/day)	r = − 0.15 p = 0.23	**r = **− **0.44** **p = 0.0001**
Total sleep time (h/day)	r = − 0.14 p = 0.26	**r = **− **0.44** **p < 0.0001**
Sleep efficiency (%)	r = 0.08 p = 0.51	r = − 0.09 p = 0.42
Sleep fragmentation index (%)	r = 0.17 p = 0.17	r = 0.09 p = 0.42

Spearman correlation coefficients. Significant correlations in bold

Correlations between sleep and physical activity measures in adults are show in Table 4. In adults, average daily duration of prolonged sedentary time was significantly negatively correlated with time in bed and total sleep time, but not sleep efficiency or sleep fragmentation index. The average duration of prolonged sedentary bouts was significantly positively correlated with sleep efficiency in adults. Adults meeting sleep recommendations had significantly less prolonged sedentary time than adults not meeting sleep recommendations (daily median duration of 174 min versus 239 min, Wilcoxon p = 0.007). There were no other significant differences in physical activity in adults meeting versus not meeting sleep recommendations.

**Table 4:** Correlations between sleep and physical activity in adults

Adults (n = 71)	Time in bed (h/day)	Total sleep time (h/day)	Sleep efficiency (%)	Sleep fragmentation index (%)
Median duration of prolonged sedentary bouts (min)	r = 0.03 p = 0.80	r = 0.05 p = 0.69	**r = 0.24** **p = 0.048**	r = − 0.23 p = 0.06
Total prolonged sedentary bout time (min/day)	**r = **− **0.32** **p = 0.009**	**r = **− **0.28** **p = 0.02**	r = − 0.04 p = 0.75	r = − 0.07 p = 0.59
Total activity Z-score (standard deviations)	r = 0.15 p = 0.24	r = 0.12267 p = 0.319	r = 0.04 p = 0.77	r = − 0.13 p = 0.28

Spearman correlation coefficients. Significant correlations in bold

Correlations between sleep and physical activity measures in all children are shown in Table 5. The average daily duration of prolonged sedentary time was significantly correlated with all measures of sleep duration and quality in children. Neither the average duration of prolonged sedentary bouts or total activity z-score was significantly correlated with measures of sleep in children. There were no significant differences in physical activity in children meeting versus not meeting sleep recommendations.

**Table 5:** Correlations between sleep and physical activity in all children 6–18 years

Children (n = 82)	Time in bed (h/day)	Total sleep time (h/day)	Sleep efficiency (%)	Sleep fragmentation index (%)
Median duration of prolonged sedentary bouts (min)	r = 0.12 p = 0.29	r = 0.14 p = 0.22	r = 0.16 p = 0.18	r = − 0.06095 p = 0.6060
Total prolonged sedentary bout time (min/day)	**r = **− **0.52** **p < 0.0001**	**r = **− **0.52** **p < 0.0001**	r = − 0.19 p = 0.11	**r = 0.33** **p = 0.004**
Total activity Z-score (standard deviations)	r = 0.20 p = 0.09	r = 0.20 p = 0.10	r = 0.06 p = 0.60	r = − 0.20 p = 0.09

Spearman correlation coefficients. Significant correlations in bold

Correlations between sleep and activity measures in children age 6–12 years are shown in Table 6. The median duration of prolonged sedentary bouts was significantly positively correlated with sleep efficiency. The average daily duration of prolonged sedentary time was significantly negatively correlated with average sleep duration and time in bed, and significantly positively correlated with sleep fragmentation index. Total activity z-score was significantly positively correlated with average sleep duration and time in bed. Correlations between sleep and activity measures in children age 13–18 years are shown in Table 7. Only average daily duration of prolonged sedentary time was significantly negatively correlated with sleep duration, both average sleep duration and time in bed.

**Table 6:** Correlations between sleep and physical activity in children 6–12 years

Children (n = 39)	Time in bed (h/day)	Total sleep time (h/day)	Sleep efficiency (%)	Sleep fragmentation index (%)
Median duration of prolonged sedentary bouts (min)	r = 0.20 p = 0.24	r = 0.27 p = 0.11	**r = 0.45** **p = 0.006**	r = − 0.23 p = 0.17
Total prolonged sedentary bout time (min/day)	**r = **− **0.51** **p = 0.001**	**r = **− **0.50** **p = 0.002**	r = − 0.19 p = 0.28	**r = 0.42** **p = 0.01**
Total activity Z-score (standard deviations)	**r = 0.36** **p = 0.03**	**r = 0.36** **p = 0.03**	r = 0.20 p = 0.24	r = − 0.19 p = 0.26

Spearman correlation coefficients. Significant correlations in bold

**Table 7:** Correlations between sleep and physical activity in children 13–18 years

Children (n = 38)	Time in bed (h/day)	Total sleep time (h/day)	Sleep efficiency (%)	Sleep fragmentation Index (%)
Median duration of prolonged sedentary bouts (min)	r = − 0.06 p = 0.74	r = − 0.07 p = 0.69	r = − 0.05 p = 0.79	r = 0.12 p = 0.48
Total prolonged sedentary bout time (min/day)	**r = **− **0.46** **p = 0.005**	**r = **− **0.48** **p = 0.003**	r = − 0.12 p = 0.48	r = 0.30 p = 0.08
Total activity Z-score (standard deviations)	r = 0.10 p = 0.55	r = 0.13 p = 0.47	r = − 0.10 p = 0.55	r = 0.21 p = 0.23

Spearman correlation coefficients. Significant correlations in bold

## Discussion

Sleep and physical activity are important components of obesity prevention, and we evaluated these behaviors in the largest cohort to date of children and adults with BBS, a population where obesity is ubiquitous. Our findings indicated that adults with BBS spent less time in bed and slept for a shorter duration than children, as would be expected in the general population, also. When examined as a percentage of the American Academy of Sleep Medicine age-recommended sleep time [13, 14], adults were clearly less apt to get as much sleep as they should, but over one in five children with BBS also got less than the amount of sleep recommended for their age. Failure to accumulate the age-recommended amount of sleep was very common in BBS, as only 10% of adults and 7% of children got adequate sleep. In population-based surveys approximately 60% of children over the age of 6 years achieve adequate sleep durations [16], and a similar percentage of adults achieve adequate sleep durations [17]. Short sleep duration is a risk factor for higher increases in BMI z-scores in young children [6]. It is unclear how sleep duration is related to weight patterns in people with BBS and should be investigated further. These findings suggest that interventions aimed at increasing the frequency of achieving age-recommended sleep times appear warranted in people of all ages with BBS. Future studies to evaluate the impact of sleep duration and meeting sleep recommendations on obesity, and related conditions like type 2 diabetes mellitus and sleep apnea are warranted.

While sleep quantity measures were less than optimal, sleep continuity appeared good in adults. Optimal sleep efficiency is above 85% in adults [18], and over 90% adults had a sleep efficiency above 85%. Optimal sleep efficiency in children is an efficiency of 90% or greater, and only 55% of children overall met this target. Sleep efficiency was particularly suboptimal in children age 13–18, with only 47% meeting sleep efficiency targets. Sleep efficiency is negatively correlated with age in other populations [19], and the trend is similar in children with BBS. The impact of suboptimal sleep efficiency in children, and particularly in adolescents, with BBS merits further investigation, including a better understanding of determinants as well as strategies to improve sleep efficiency. Sleep efficiency and sleep fragmentation can be impacted by sleep apnea. In our cohort, 28% of adults and 10% of children were recommended treatment for sleep apnea by self-report and 35% and 26% respectively reported having an abnormal sleep study. Future research in this population should evaluate clinically diagnosed sleep apnea as well as adherence to treatment on sleep efficiency and sleep fragmentation.

Visual impairment is common in BBS, and in other populations with vision impairment the degree and severity of impairment has adverse impacts on physical activity levels [20]. In BBS, vision impairment is progressive with age; people with BBS are often very severely visually impaired by early adolescence [21, 22]. This may at least partially explain the negative relationships between total activity score and age.

Interventions targeting sleep behavior also have the potential of impacting physical activity behavior and vice versa. Large cohort studies have described associations between physical activity and sleep behavior [23]. Sleep duration and sedentary behavior have been shown to have a bidirectional relationship; short sleep duration is associated with more sedentary time [24]. In a study of people with retinitis pigmentosa, greater wake after sleep onset, a measure of sleep continuity, was associated with lower daytime activity level [25]. We found more consistent associations between measures of sleep duration and sedentary time, although both measures of sleep duration and sleep continuity were associated with the duration of prolonged sedentary time in children. Children with more prolonged sedentary time had significantly lower sleep duration, lower sleep efficiency, and higher sleep fragmentation. In adults, more prolonged sedentary time was also negatively correlated with time in bed and total sleep time. Adults spent almost twice as much total time engaged in prolonged sedentary behavior as children, but children engaged in longer average bouts of prolonged sedentary behavior.

Both adults and children with BBS would likely benefit from interventions designed to disrupt sedentary time. Poor sleep is a known risk for chronic kidney disease and increased cardiovascular risk [26–30], leading causes of premature death in BBS. Our findings support the importance of effective activity and sleep interventions in this high risk patient population.

Key strengths of our study include a relatively large cohort of people with BBS. We included both children and adult participants to gain a better understanding of physical activity and sleep patterns across the lifespan in people with BBS. We also used a 12-day measurement period for the objective assessment of activity using accelerometry. Objective methods are not prone to recall bias which is an advantage over self-report tools.

A limitation of our study is that, while wrist-worn accelerometers have been repeatedly shown to demonstrate good agreement with sleep parameters measured by polysomnography, less is known about the agreement in populations where sleep disordered breathing or sleep apnea may be more common. Of the studies that have compared wrist-worn accelerometer sleep parameters with polysomnography, the agreement has been good [31]. We did not have a parallel group of matched, BBS-free participants to directly compare our sleep and physical activity results to. Additionally, over 95% of children with BBS have overweight or obesity by age 6 [3]. We only had five children age 3–5 years participate in our study, and we were unable to include this age group in our analysis. This is an important age group to target for overweight or obesity prevention and treatment.

Our findings identified important behavioral targets to potentially help prevent or mitigate obesity in individuals with BBS. Insufficient sleep duration was common in both children and adults with BBS, as was prolonged sedentary bouts. Identifying approaches to increase sleep duration and decrease prolonged sedentary time have the potential to enhance health and well-being in people with BBS of all ages.

## Supplementary Information

**Additional file 1.** Scatterplots of Spearman correlations between measures of sleep and measures of physical activity in children with BBS. Figure 1A shows comparisons between children age 6-12 and Figure 1B shows children 13-18. Consistent with Spearman correlations data are showns as ranks rather than raw values for sleep and physical activity variables.

## Data Availability

The data generated during this study are contained in this published article.

## References

[1] Haws RM, Krentz AD, Stankowski RV, Steiner RD (2015). Bardet–Biedl syndrome: a model for translational research in rare diseases. New Horizons Transl Med.

[2] Hamlington B, Ivey LE, Brenna E, Biesecker LG, Biesecker BB, Sapp JC (2015). Characterization of courtesy stigma perceived by parents of overweight children with Bardet–Biedl syndrome. PLoS ONE.

[3] Pomeroy J, Krentz AD, Richardson JG, Berg RL, VanWormer JJ, Haws RM (2021). Bardet–Biedl syndrome: weight patterns and genetics in a rare obesity syndrome. Pediatr Obes.

[4] Yeung JC, Katwa UA, Lee GS (2017). Sleep disordered breathing in Bardet–Biedl syndrome. Int J Pediatr Otorhinolaryngol.

[5] Grace C, Beales P, Summerbell C, Jebb SA, Wright A, Parker D, Kopelman P (2003). Energy metabolism in Bardet–Biedl syndrome. Int J Obes Relat Metab Disord.

[6] Xiu L, Ekstedt M, Hagströmer M, Bruni O, Bergqvist-Norén L, Marcus C (2020). Sleep and adiposity in children from 2 to 6 years of age. Pediatrics.

[7] Piirtola M, Kaprio J, Svedberg P, Silventoinen K, Ropponen A (2020). Associations of sitting time with leisure-time physical inactivity, education, and body mass index change. Scand J Med Sci Sports.

[8] Bowden Davies KA, Sprung VS, Norman JA (2019). Physical activity and sedentary time: association with metabolic health and liver fat. Med Sci Sports Exerc.

[9] Kakutani-Hatayama M, Kadoya M, Morimoto A (2020). Associations of sleep quality, sleep apnea and autonomic function with insulin secretion and sensitivity: HSCAA study. Metabol Open.

[10] ActiGraph. Knowledge. How does sedentary analysis work? November 21, 2020. https://actigraphcorp.force.com/support/s/article/How-does-Sedentary-Analysis-work.

[11] Sadeh A, Sharkey KM, Carskadon MA (1994). Activity-based sleep-wake identification: an empirical test of methodological issues. Sleep.

[12] Cole RJ, Kripke DF, Gruen W, Mullaney DJ, Gillin JC (1992). Automatic sleep/wake identification from wrist activity. Sleep.

[13] Paruthi S, Brooks LJ, D'Ambrosio C (2016). Consensus statement of the american academy of sleep medicine on the recommended amount of sleep for healthy children: methodology and discussion. J Clin Sleep Med.

[14] Watson NF, Badr MS, Belenky G (2015). Recommended amount of sleep for a healthy adult: a joint consensus statement of the American Academy of Sleep Medicine and Sleep Research Society. Sleep.

[15] ActiGraph. Documentation. Sleep Fragmentation Index – Validation reference. November 8, 2018. https://actigraphcorp.force.com/support/s/article/Sleep-Fragmentation-Index-Validation-Reference.

[16] Tsao HS, Gjelsvik A, Sojar S, Amanullah S (2021). Sounding the alarm on sleep: a negative association between inadequate sleep and flourishing. J Pediatr.

[17] Centers for Disease Control and Prevention. National Center for Health Statistics. National Health and Nutrition Examination Survey (NHANES). About the National Health and Nutrition Examination Survey. https://www.cdc.gov/nchs/nhanes/about_nhanes.htm. Accessed on 15 Nov 2018.

[18] Ohayon M, Wickwire EM, Hirshkowitz M (2017). National Sleep Foundation's sleep quality recommendations: first report. Sleep Health.

[19] Ohayon MM, Carskadon MA, Guilleminault C, Vitiello MV (2004). Meta-analysis of quantitative sleep parameters from childhood to old age in healthy individuals: developing normative sleep values across the human lifespan. Sleep.

[20] Ong SR, Crowston JG, Loprinzi PD, Ramulu PY (2018). Physical activity, visual impairment, and eye disease. Eye (Lond).

[21] Fulton AB, Hansen RM, Glynn RJ (1993). Natural course of visual functions in the Bardet–Biedl syndrome. Arch Ophthalmol.

[22] Berezovsky A, Rocha DM, Sacai PY, Watanabe SS, Cavascan NN, Salomão SR (2012). Visual acuity and retinal function in patients with Bardet–Biedl syndrome. Clinics (Sao Paulo).

[23] Kim Y, Wijndaele K, Sharp SJ (2019). Specific physical activities, sedentary behaviours and sleep as long-term predictors of accelerometer-measured physical activity in 91,648 adults: a prospective cohort study. Int J Behav Nutr Phys Act.

[24] Lin Y, Tremblay MS, Katzmarzyk PT (2018). Temporal and bi-directional associations between sleep duration and physical activity/sedentary time in children: an international comparison. Prev Med.

[25] Bittner AK, Haythornthwaite JA, Patel C, Smith MT (2018). Subjective and objective measures of daytime activity and sleep disturbance in retinitis pigmentosa. Optom Vis Sci.

[26] McMullan CJ, Curhan GC, Forman JP (2016). Association of short sleep duration and rapid decline in renal function. Kidney Int.

[27] Ricardo AC, Knutson K, Chen J (2017). The association of sleep duration and quality with CKD progression. J Am Soc Nephrol.

[28] Yamamoto R, Shinzawa M, Isaka Y (2018). Sleep quality and sleep duration with CKD are associated with progression to ESKD. Clin J Am Soc Nephrol.

[29] Daghlas I, Dashti HS, Lane J (2019). Sleep duration and myocardial infarction. J Am Coll Cardiol.

[30] Park S, Lee S, Kim Y (2020). Short or long sleep duration and CKD: a Mendelian randomization study. J Am Soc Nephrol.

[31] Giménez S, Videla L, Romero S (2018). Prevalence of sleep disorders in adults with down syndrome: a comparative study of self-reported, actigraphic, and polysomnographic findings. J Clin Sleep Med.

