# Supplementary material for: Sleep and physical activity patterns in adults and children with Bardet–Biedl syndrome
Source: Orphanet J Rare Dis. 2021 Jun 14;16:276. doi: 10.1186/s13023-021-01911-4 (PMC8201861; doi:10.1186/s13023-021-01911-4)
Supplement: Supplementary file 1 — Additional file 1. Scatterplots of Spearman correlations between measures of sleep and measures of physical activity in children with BBS. Figure 1A shows comparisons between children age 6-12 and Figure 1B shows children 13-18. Consistent with Spearman correlations data are showns as ranks rather than raw values for sleep and physical activity variables. [file 13023_2021_1911_MOESM1_ESM.pptx]

## Slide 1
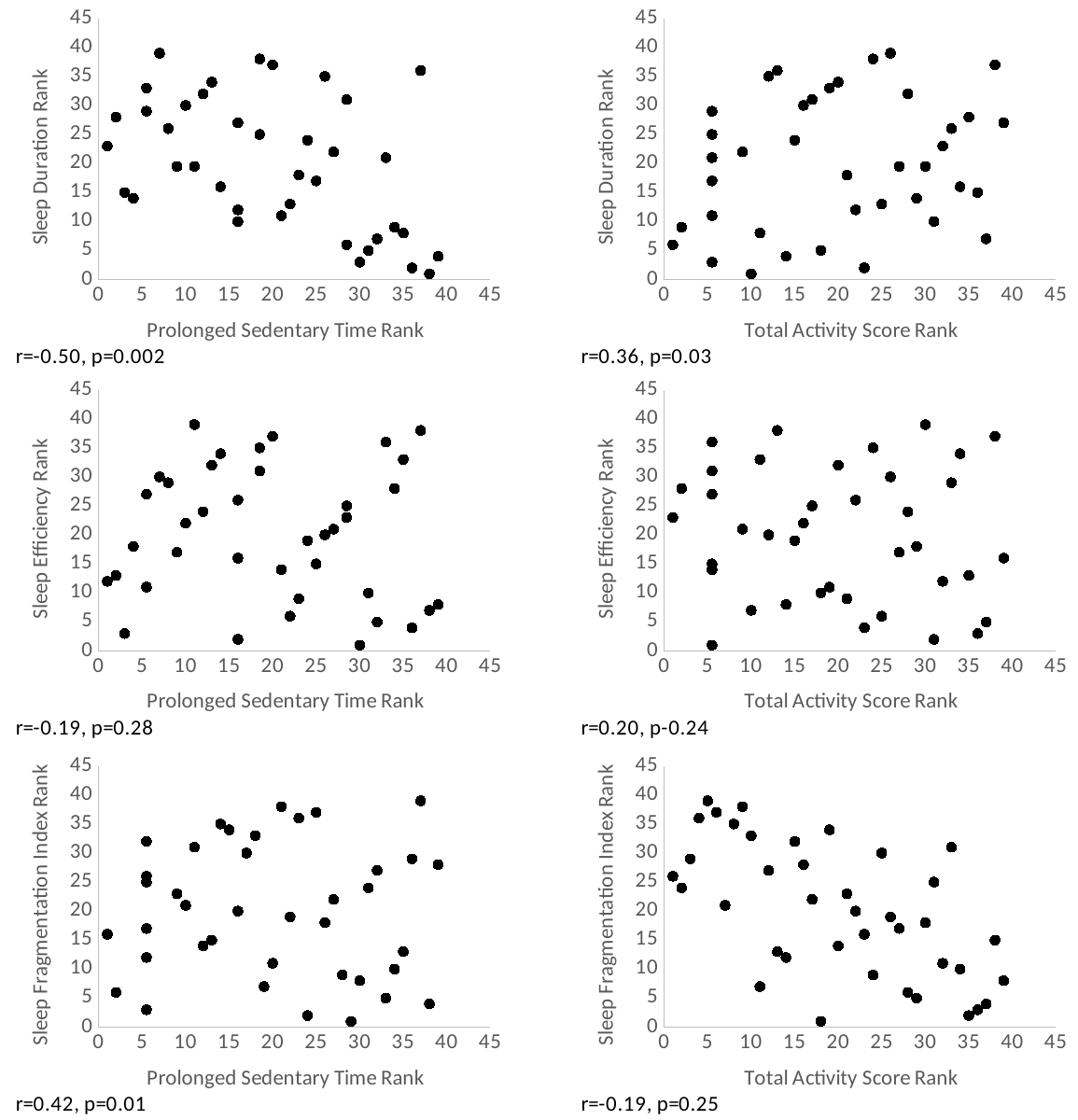

### Chart
| Category | |
|---|---|
### Chart
| Category | |
|---|---|
### Chart
| Category | |
|---|---|
### Chart
| Category | |
|---|---|
### Chart
| Category | |
|---|---|
### Chart
| Category | |
|---|---|

## Slide 2
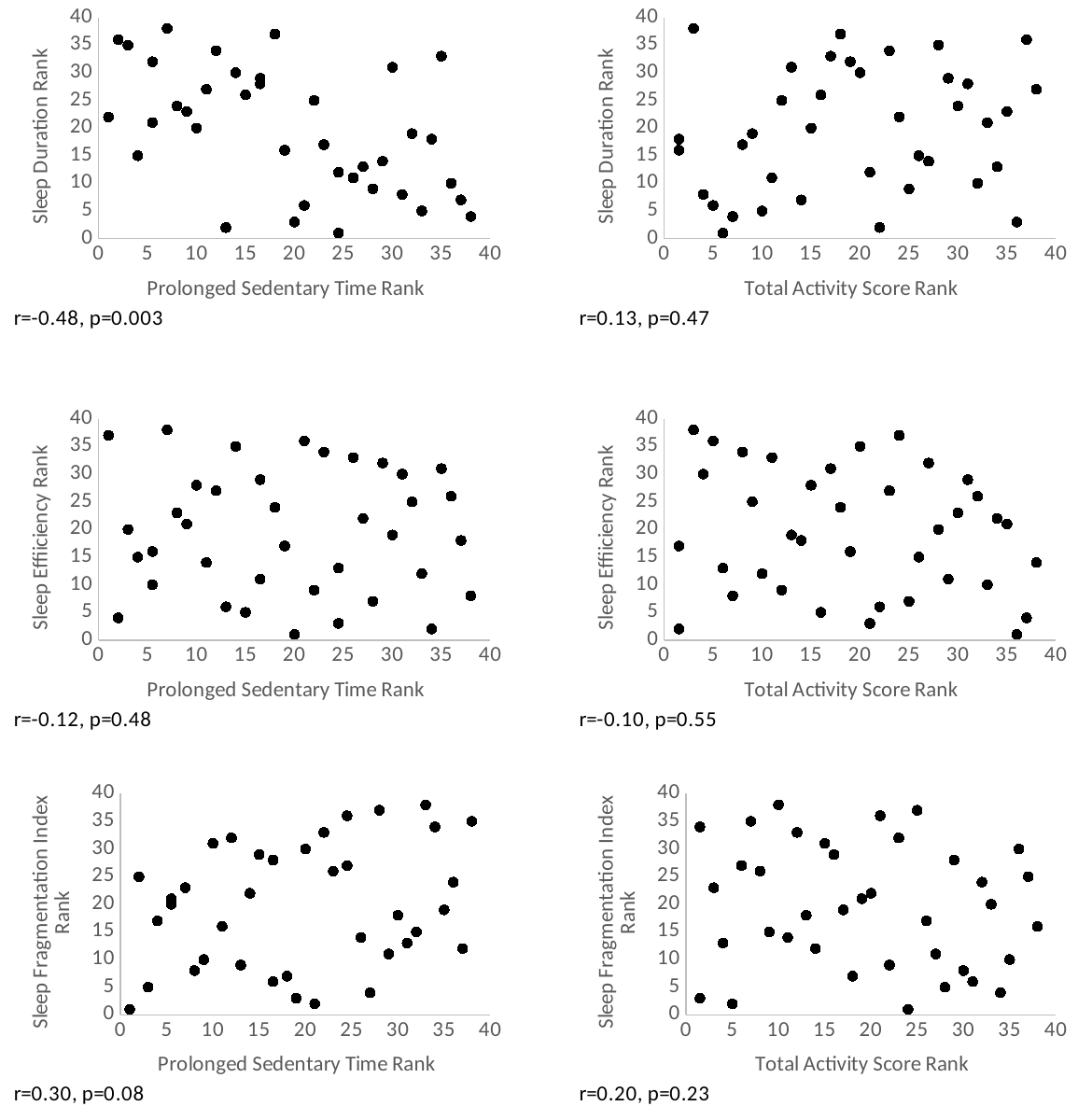

### Chart
| Category | |
|---|---|
### Chart
| Category | |
|---|---|
### Chart
| Category | |
|---|---|
### Chart
| Category | |
|---|---|
### Chart
| Category | |
|---|---|
### Chart
| Category | |
|---|---|
